# Amyloid fibrils degradation: the pathway to recovery or aggravation of the disease?

**DOI:** 10.3389/fmolb.2023.1208059

**Published:** 2023-06-12

**Authors:** Maksim I. Sulatsky, Olga V. Stepanenko, Olesya V. Stepanenko, Ekaterina V. Mikhailova, Irina M. Kuznetsova, Konstantin K. Turoverov, Anna I. Sulatskaya

**Affiliations:** ^1^ Laboratory of cell morphology, Institute of Cytology of the Russian Academy of Sciences, St. Petersburg, Russia; ^2^ Laboratory of structural dynamics, stability and folding of proteins, Institute of Cytology of the Russian Academy of Sciences, St. Petersburg, Russia

**Keywords:** amyloidosis, alzheimer’s disease, amyloids’ degradation, cytotoxicity, superfolder green fluorescent protein (sfGFP), Aβ-peptide (Aβ42)

## Abstract

**Background:** The most obvious manifestation of amyloidoses is the accumulation of amyloid fibrils as plaques in tissues and organs, which always leads to a noticeable deterioration in the patients’ condition and is the main marker of the disease. For this reason, early diagnosis of amyloidosis is difficult, and inhibition of fibrillogenesis, when mature amyloids are already accumulated in large quantities, is ineffective. A new direction for amyloidosis treatment is the development of approaches aimed at the degradation of mature amyloid fibrils. In the present work, we investigated possible consequences of amyloid’s degradation.

**Methods:** We analyzed the size and morphology of amyloid degradation products by transmission and confocal laser scanning microscopy, their secondary structure and spectral properties of aromatic amino acids, intrinsic chromophore sfGFP, and fibril-bound amyloid-specific probe thioflavin T (ThT) by the absorption, fluorescence and circular dichroism spectroscopy, as well as the cytotoxicity of the formed protein aggregates by MTT-test and their resistance to ionic detergents and boiling by SDS-PAGE.

**Results:** On the example of sfGFP fibrils (model fibrils, structural rearrangements of which can be detected by a specific change in the spectral properties of their chromophore), and pathological Aβ-peptide (Aβ42) fibrils, leading to neuronal death in Alzheimer’s disease, the possible mechanisms of amyloids degradation after exposure to factors of different nature (proteins with chaperone and protease activity, denaturant, and ultrasound) was demonstrated. Our study shows that, regardless of the method of fibril degradation, the resulting species retain some amyloid’s properties, including cytotoxicity, which may even be higher than that of intact amyloids.

**Conclusion:** The results of our work indicate that the degradation of amyloid fibrils *in vivo* should be treated with caution since such an approach can lead not to recovery, but to aggravation of the disease.

## 1 Introduction

Amyloidosis is a serious disease associated with the formation of pathological amyloid fibrils, their accumulation in tissues and organs of humans and animals, and the occurrence of amyloid fibril clots, the so-called plaques. To date, several dozens of such disorders have been identified (for example, systemic lysozyme, insulin, hemodialysis amyloidosis, and many neurodegenerative diseases, such as Alzheimer’s, Parkinson’s, prion diseases, *etc.*) (Khan and Falk, 2001; Westermark et al., 2005; Chiti and Dobson, 2006; Dobson, 2006; Rambaran and Serpell, 2008; Eisenberg and Jucker, 2012; Soto and Pritzkow, 2018; Gertz and Dispenzieri, 2020). These diseases are going become epidemic soon, given the increase in life expectancy (Licastro et al., 2014; Patterson, 2018). The impact of amyloids on the body can manifest itself as an isolated lesion of one (local amyloidosis) or several (systemic amyloidosis) organs at once, triggering a whole cascade of pathological processes (Sipe et al., 2016). The formation of amyloid plaques is the most obvious manifestation of the disease, always leads to a marked deterioration in the condition of patients and is the main marker of the disease. Amyloidosis are diagnosed only when many amyloid plaques have already accumulated in the body and the potential anti-amyloid drugs aimed at inhibiting fibrillogenesis are not effective. Given this fact, the removal of already formed amyloids from the body is currently considered as one of the most promising approaches to amyloidosis treatment (Wechalekar et al., 2016; Rysava, 2019).

Surgical removal of amyloid plagues often shows its inefficiency, since the disease can recur, and repeated surgical interventions pose a risk of patient disability. In this regard, detection and direct degradation of amyloids *in vivo* is currently considered a more applicable strategy for their removal from the body (Sevigny et al., 2016; Khan et al., 2020). In particular, the “Aducanumab” drug for the treatment of Alzheimer’s disease approved by the FDA (US Food and Drug Administration) at the beginning of 2021, is an antibody to amyloids formed from Abeta-peptide, that promotes their subsequent destruction by cells of the immune system (Dhillon, 2021). Three more drugs with a similar mechanism of action are currently in clinical trials (https://www.clinicaltrialsarena.com/analysis/alzheimers-disease-clinical-trials/). However, already at the end of 2021, there was a report of serious side effects of the “Aducanumab” (in more than 40% of cases), manifested in cerebral edema and microhemorrhages (Salloway et al., 2022). Such an ambiguous and controversial clinical effect is largely due to a significant gap in the existing understandings about the properties of the species formed during the degradation of amyloids and consequences of this process for cells and tissues. The relevance of this problem is confirmed by intensive research focusing on the analysis of the effects of individual substances and factors on amyloid fibrils formed from various proteins and peptides (Emeson et al., 1966; Soderberg et al., 2005; Chander et al., 2006; Shin et al., 2008; Giorgetti et al., 2011; Azevedo et al., 2012; Gao et al., 2015; Poepsel et al., 2015; Yagi et al., 2015; Stepanenko et al., 2020; Sulatsky et al., 2020; Stepanenko et al., 2021a). However, comprehensive study of the influence of a wide range of factors with different mechanisms of action on amyloids formed from the same protein has not been carried out. Such a study is necessary to get a general idea of the properties of protein species formed as a result of amyloid degradation.

To this aim, in the present work using a wide range of modern spectroscopic, microscopic, chromatographic, biochemical, and cytological methods (including specially developed ones) we analyze the influence of several external factors of various nature (proteins with chaperone and protease activity, denaturant, and ultrasound) on the size, morphology, secondary structure, resistance to ionic detergents and temperature, as well as cytotoxicity of mature amyloids. We choose as an object newly identified amyloid fibrils formed from the widely used fluorescent biomarker super folder GFP (sfGFP) (Pedelacq et al., 2006). Some factors make sfGFP amyloids an attractive object for research, including the low cost of the recombinant protein (or ease of its isolation and purification in the amounts required for the intended analysis), the ease of sfGFP amyloids preparation, and the good reproducibility of the structure and properties of sfGFP fibrils. The additional advantage of sfGFP amyloids is the possibility of detecting structural rearrangements in these fibrils by a specific change in the spectral properties of their chromophore. The sfGFP aggregates have all the properties that are characteristic of *bona fide* amyloid: fibrous structure enriched with β-sheets, high resistance to ionic detergents, ability to bind to the amyloid-specific ThT probe, and high cytotoxicity (Stepanenko et al., 2021b; Sulatskaya et al., 2022). This means that the data on the mechanisms of amyloid degradation obtained for sfGFP fibrils can be extrapolated to pathological amyloids. To confirm the universality of the results obtained for sfGFP amyloids a limited range of studies (analysis of size, morphology and cytotoxicity) after similar exposures was performed for Aβ-peptide (Aβ42) amyloids, which are a hallmark of Alzheimer’s disease (Masters and Selkoe, 2012; Wang et al., 2016).

## 2 Materials and methods

### 2.1 Materials

Fluorescent dye thioflavin T (ThT) “UltraPure Grade” from AnaSpec (United States ), Aβ42 peptide from GL Biochem (China), 1,1,1,3,3,3-Hexafluoro-2-propanol (HFIP), 3-(4,5-dimethylthiazol-2-yl)-2,5-diphenyltetrazolium bromide (MTT), guanidine hydrochloride (GdnHCl), trypsin from bovine pancreas, and buffer components from Sigma (United States ), isopropyl-beta-D-1-thiogalactopyranoside (IPTG) from Fluka (Switzerland) were used without additional purification. Reagents for cell cultivation, including DMEM medium (glucose 4.5 g/L), fetal bovine serum (FBS), and 0.25% Trypsin-EDTA were acquired from Gibco (Thermo Fisher Scientific, United States ). Culture flasks and 96-well plates (flat bottom) were purchased from Corning (United States ).

### 2.2 Gene expression and protein purification

The plasmid pET-28a (+) encoding for superfolder GFP (Pedelacq et al., 2006) with poly-histidine tag was constructed as described previously (Mishin et al., 2008) and was transformed into an *Escherichia coli BL21(DE3)* host. The sfGFP expression was induced by incubation of the cells with 0.5 mM IPTG for 24 h at 23 °C. The recombinant protein was purified with affinity chromatography on His- GraviTrap columns packed with Ni^2+^ -agarose (GE Healthcare, Sweden). The purity of the protein was not less than 95%, as indicated by SDS-PAGE in 15% polyacrylamide gel (Laemmli, 1970).

The plasmid pET-28a (+) encoding for alpha-B-crystallin was constructed as described previously (Stepanenko et al., 2020) and was transformed into an *E. coli* BL21 (DE3) host. The expression of alpha-B-crystallin was initiated by 0.5 mM IPTG at 28 °C for 24 h. Alpha-B-crystallin isolation and purification was performed as described previously (Mymrikov et al., 2010; Datskevich et al., 2012). The purified protein was highly concentrated (up to 6–10 mg/mL) and stored in 25 mM TrisHCl, 0.1 mM EDTA, 50 mM NaCl, 0.1 mM PMSF, 1 mM DTT, pH 7.6. The purity of the protein was tested by SDS-PAGE in 12% polyacrylamide gels (Laemmli, 1970). The protein concentration was calculated using an extinction coefficient at 280 nm of 14,100 M^−1^ cm^−1^ according to amino acids composition.

### 2.3 Amyloid fibrils preparation and degradation

Amyloid fibrils of sfGFP were prepared by protein incubation 20 mM NaH_2_PO_4_/Na_2_HPO_4_ (pH 7.4) buffer at 57 °C for 2 weeks as previously described (Stepanenko et al., 2021b; Sulatskaya et al., 2022). The protein concentration was 2 mg/mL. Aβ42 were dissolved in 50% organic solvent 1,1,1,3,3,3-Hexafluoro-2-propanol (HFIP, final concentration was 1 mg/mL) and incubated for 7 days (Kayed et al., 2007; Broersen et al., 2011). Afterward, the HFIP was slowly evaporated under a stream of nitrogen, then the volume of the sample was adjusted with distilled water to the initial one, and the samples were incubated for an additional 7 days. Amyloid fibrils were grown at constant agitation (500 rpm). The constant temperature was maintained by TS-100 Thermo-Shaker (Biosan, United States ).

Degradation of amyloid fibrils was induced by their incubation in the presence of GdnHCl (at a final concentration of 6 M), trypsin (at a weight ratio of enzyme to fibrils of 1/125), and alpha-B-crystallin (at a molar ratio of enzyme to fibrils of 0.5/1) for 5 days. The concentration of GdnHCl (Narimoto et al., 2004; Sulatsky et al., 2020), trypsin (Monti et al., 2005; Myers et al., 2006; Relini et al., 2008; Stepanenko et al., 2021a), and alpha-B-crystallin (Rekas et al., 2007; Shammas et al., 2011; Esposito et al., 2013; Stepanenko et al., 2020) for the experiment were chosen based on the literature. The incubation time was chosen based on the data from our previous studies, showing that this time is sufficient to complete all structural changes in the fibrils under the influence of the selected factors (Stepanenko et al., 2020; Sulatsky et al., 2020; Stepanenko et al., 2021a). Freshly thawed enzymes were used. Trypsin stock was stored in acidic conditions (1 mM HCl) to avoid autolysis (Sipos and Merkel, 1970; Walsh, 1970). Trypsin proteolysis of fibrils was quenched with 0.1 mM phenylmethylsulfonyl fluoride (PMSF). The GdnHCl concentration was controlled using an Abbe refractometer (LOMO, Russia). Amyloid fibrils were also degraded by ultrasonication for 5 min at 37 °C in a water bath-type ultrasonic transmitter Elmasonic P30H with a temperature controller (Elma GmbH, Germany). The volume of the water bath was about 2.5 L. The instrument frequency was 37 kHz, and the power output was set to deliver a maximum of 400 W. Samples were ultrasonicated from three directions (i.e., two sides and bottom).

### 2.4 Transmission electron microscopy

A transmission electron microscope Libra 120 (Carl Zeiss, Germany) was applied to produce the micrographs of amyloid fibrils and their degradation products. Samples were put on the copper grids coated with formvar/carbonoscopy films (Electron Microscopy Sciences, United States ) and stained by a 1% aqueous solution of uranyl acetate.

### 2.5 Spectral measurements

A U-3900H spectrophotometer (Hitachi, Japan) was applied to collect the absorption spectra of the samples. The absorption spectra of all samples and mixtures of samples with ThT were corrected by the light scattering according to the standard procedure (Vladimirov and Litvin, 1964). The concentration of sfGFP and ThT was quantified using molar extinction coefficients of ε_280_ = 31,519 M^-1^cm^-1^ and ε_412_ = 31,600 M^-1^cm^-1^, respectively. The final concentration of fibril used for spectroscopic measurement was 0.4 mg/mL in all cases except for study by circular dichroism where it was 0.15 mg/mL. The absorbance of a dye did not exceed 0.5. The size polymorphic species of sfGFP amyloids after their degradation was estimated by turbidity recorded at 550 nm and Rayleigh light scattering recorded at 295 nm (λ_ex_ = 295 nm).

Fluorescence spectra of the sfGFP amyloid samples were measured using a Cary Eclipse spectrofluorimeter (Varian, Australia). Fluorescence of internal fluorophores of sfGFP amyloids, including a single tryptophan Trp57, tyrosine residues and a chromophore, was excited at a wavelength of 280 nm. Fluorescence of ThT was excited at a wavelength of 440 nm and recorded at a wavelength of 480 nm. As both the chromophore of sfGFP amyloids and ThT are excited at a wavelength of 440 nm, the sample of sfGFP amyloids alone was used as a blank for the sample of sfGFP amyloids in the presence of ThT. The spectral slits width did not exceed 5 nm in all experiments. The intrinsic fluorescence of sfGFP amyloids and ThT fluorescence intensity were corrected on the primary inner filter effect (Fonin et al., 2014; Sulatskaya et al., 2017a).

Fluorescence decay curves of ThT bound to studied polymorphic species were collected using a spectrometer FluoTime 300 (PicoQuant, Germany) with the Laser Diode Head LDH-C-440 (λ_ex_ = 440 nm). The fitting of fluorescence decay curves was performed using the standard convolute-and-compare nonlinear least-squares procedure (O’Connor, 1984) with minimization according to Marquardt (Marquardt, 1963).

Far-UV CD spectra were measured using a J-810 spectropolarimeter (Jasco, Japan). The far-UV CD spectra were recorded in a 1 mm path length cell from 260 to 190 nm. Corrected CD spectra were obtained as the average of three scans with the subtraction of the buffer solution baseline. The secondary structure content of sfGFP amyloids was estimated using their corrected CD spectra by the BeStSel webserver (Micsonai et al., 2018) which has the advantage over the previously available methods in that it is able to assess the β-sheet-rich structure of protein aggregates and amyloid fibrils (Wang et al., 2021). In case of such samples, spectral artifacts caused by differential light scattering, precipitation, or linear dichroism can affect or interfere with an accurate secondary structure analysis (Tinoco et al., 1980). Therefore, we used a relatively low sample concentration (0.15 mg/mL) to ensure that the samples measured were homogenous solutions without large insoluble precipitations. It was controlled that analyzed CD spectra after a proper baseline subtraction contained no significant residual signal in the wavelength region of 250–260 nm. This indicated that the recorded spectra were not distorted by light scattering.

### 2.6 Sodium dodecyl sulfate (SDS) and pseudo-native SDS gel electrophoresis

Pseudo-native SDS-PAGE analysis of sfGFP amyloid samples (that according to the literature and our earlier results, provide the detection of oligomers of GFP and its homologs in the sample (Wiedenmann et al., 2002; Yanushevich et al., 2002)) at a concentration of 0.4 mg/mL was performed on 17% and 8% polyacrylamide gel (0.375 M Tris HCl, pH 8.8, 0.1% SDS). Samples were loaded on the gel in a buffer containing 0.0625 M Tris HCl, pH 6.8, 1% SDS, 10% glycerol, and 0.002% bromophenol blue without boiling.

Native PAGE analysis of Aβ42 amyloid samples at a concentration of 0.4 mg/mL was performed on 17% polyacrylamide gel (0.375 M Tris HCl, pH 8.8, 0.0375% SDS). Samples were loaded on the gel in a buffer containing 0.0625 M Tris HCl, pH 6.8, 10% glycerol, and 0.002% bromophenol blue without boiling.

SDS-PAGE analysis of sfGFP amyloid samples was performed on 17% polyacrylamide gel (0.375 M Tris HCl, pH 8.8, 0.1% SDS). Before loading on a gel, sfGFP amyloid samples were incubated in Laemmli buffer (containing 0.0625 M Tris HCl, pH 6.8, 2% SDS, 10% glycerol, 5% 2-mercaptoethanol, 0.002% bromophenol blue) and boiled for 10 min.

### 2.7 Gel filtration

Gel filtration of sfGFP samples was performed on a Superose 12 PC 3.2/30 column (GE Healthcare) using an AKTApurifier system (GE Healthcare). Protein concentration was 0.4 mg/mL. The column was pre-equilibrated in native conditions (20 mM PBS, pH 7.4) or in denaturing conditions (6 M GdnHCl in 20 mM PBS, pH 7.4) before loading the appropriate sample. A set of proteins with known molecular mass (chromatography standards from GE Healthcare) was used for column calibration under native conditions.

### 2.8 MTT assay

Human adenocarcinoma (HeLa) cells were obtained from the shared research facility “Vertebrate cell culture collection” supported by the Ministry of Science and Higher Education of the Russian Federation (Agreement No. 075-15-2021–683). Cells were cultured in DMEM medium (Gibco, United States ) supplemented with 10% FBS in a humidified incubator with 5% CO2 at 37 °C. For the experiment, HeLa cells that reached the early stationary phase were seeded on 96-well coated culture plates at a density of 6,000 viable cells per well in 100 µL of culture medium. The metabolic activity was evaluated in 24 h using an MTT inhibition assay according to standard protocol (Mosmann, 1983; Nishimura et al., 2003) as described earlier (Stepanenko et al., 2021b). Recorded data were normalized to the values of the control (cells not treated with any sample). The data obtained was the average of 6 independent measurements.

### 2.9 Statistical analysis

The spectral characteristics of sfGFP amyloid samples and ThT bound to studied polymorphic species were determined from at least three independent experiments. The standard error of the mean is determined by a confidence interval of 0.95.

When comparing the cytotoxicity in the control and experimental groups, analysis of variance (ANOVA) with *post hoc* multiple pairwise comparison according to the Tukey procedure was performed using online calculator (https://www.socscistatistics.com/tests/anova/default2.aspx). Differences were considered significant at *p* < 0.05.

## 3 Results

Amyloid fibrils formed from sfGFP were degraded by 1) denaturing agent GdnHCl (at a concentration of 6 M), 2) ultrasonication, 3) heat shock protein with chaperone-like activity alpha-B-crystallin (aBCry), 4) protein with the protease activity trypsin. The incubation time and concentration of external factors were chosen based on the literature and data from our previous studies (see Materials and methods for more details).

### 3.1 Morphology of aggregates formed after exposure to external factors on amyloid

**FIGURE 1:**
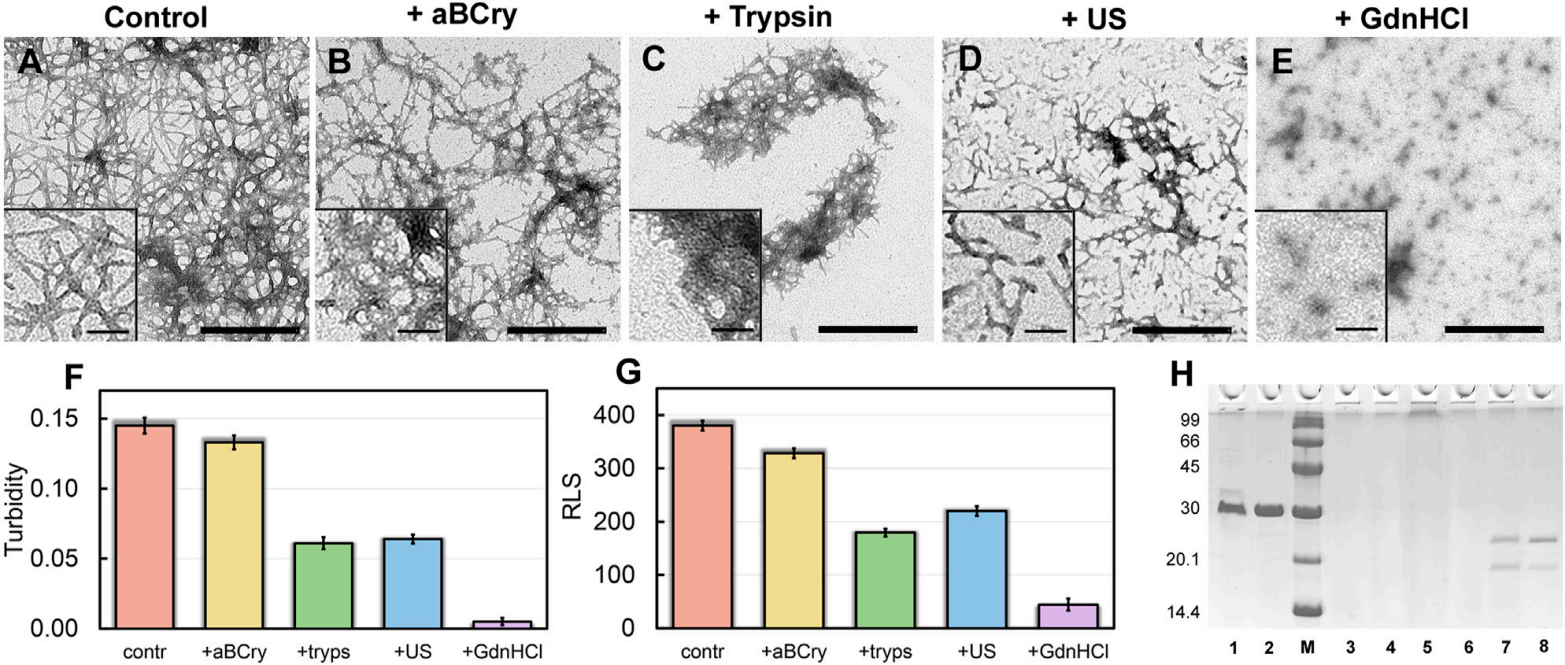
Morphology and sizes of aggregates formed after degradation of sfGFP amyloid fibrils. The electron micrographs of sfGFP aggregates **(A)** before and **(B–E)** after their treatment by **(B)** aBCry, **(C)** trypsin, **(D)** ultrasound, and **(E)** GdnHCl. The scale bars are equal to 500 nm. The (Insets) show the zoom-in images of aggregates. The scale bars are equal to 100 nm. **(F)** Turbidity and **(G)** Rayleigh Light Scattering (RLS) of the sfGFP aggregates before (contr) and after their treatment by aBCry, trypsin (tryps), ultrasound (US) and GdnHCl. **(H)** Pseudo-native SDS-PAGE (17% gel) of sfGFP aggregates before (lane 3) and after their treatment by trypsin (lane 4), GdnHCl (lane 5), ultrasound (lane 6), and aBCry (lane 7). (lane 1) and (lane 2) correspond to monomeric sfGFP before and after boiling, respectively. (lane 8) corresponds to aBCry alone. (M) corresponds to marker proteins.

Visualization of treated sfGFP amyloid samples by transmission electron microscopy (Figures 1A–E) demonstrates the sensitivity of fibrils morphology and size to external influences, despite the existing ideas about the extremely high stability of amyloids. The control intact sample contained long thin amyloid fibers capable of interacting with each other and forming bundles (Figure 1A). After the addition of aBCry, sfGFP fibrils were predominantly “fluffed up” without a noticeable decrease in their size (Figure 1B). Trypsin induced fragmentation of individual fibers and those protruding from fibrillar clusters, and only these small fragments were subjected to “fluffing” (Figure 1C). However, the dense clusters of fibers were unaffected by trypsin. Ultrasonication, on the contrary, led to the splitting of large fibrillar clots into individual fibers with their “cutting” into fragments of various sizes (Figure 1C). The later retained the morphology characteristic of amyloids. Finally, the fibrils underwent the most pronounced degradation under the action of GdnHCl: declustering of large clumps and depolymerization of individual fibers into rather small aggregates of various sizes occurred (Figure 1E). The data obtained point out at a different mechanism of amyloids’ destruction by the analyzed factors, whose influence results in a number of species of different morphology.

### 3.2 Evaluation of the efficiency of amyloid degradation

The TEM data suggest the different efficiency of sfGFP amyloid degradation induced by different factors. To confirm this assumption, we evaluated the turbidity (Figure 1F) and Rayleigh light scattering (RLS, Figure 1G) of the samples, reflecting the size and amount of protein aggregates in them. The obtained data correlate well with the TEM results. The intact sample was characterized by the highest light scattering. The recorded parameters turned out to be practically unchanged in the treatment of the sample with aBCry. This testifies, in general, to the preservation of the size/number of aggregates in the sample under the above exposure. While trypsin- and ultrasound-induced fragmentation of fibrils led to a noticeable decrease in the light scattering of the sample. The most pronounced changes in light scattering were expectedly observed in the sample after the treatment with GdnHCl. Thus, the results obtained support the different effectiveness of the impact of various factors on amyloids. At the same time, the species emerging from the processing of amyloids differ not only in morphology, but also in size.

### 3.3 Evaluation of the minimum size of amyloid degradation products and their resistance to ionic detergents

Still light scattering registration does not allow accurate quantitative estimates of the size of aggregates present in the sample. Thus, we also analyzed samples of sfGFP amyloids exposed to various factors using sodium dodecyl sulfate polyacrylamide gel electrophoresis (SDS-PAGE) in denaturing and pseudo-native conditions. Separation of amyloids’ samples treated with any of the analyzed factor on a 17% gel under pseudo-native conditions revealed no bands corresponding to protein species of molecular weight in the range from 14 to 100 kDa (Figure 1H). There were observed only bands of protein species either not entering the stacking gel or trapped on the boundary between the stacking and resolving gels, and these bands were noticeably more intense for the sample treated with GdnHCl. Separation of amyloids’ samples on 8% gel under pseudo-native conditions gave similar results (Supplementary Figure S1). Taken together our, findings indicate that GdnHCl, compared with other analyzed factors, most effectively destroy fibrils to aggregates of the smallest size, the latter, however, exceeds significantly 200 kDa. This assumption is in good agreement with the data obtained using TEM.

To verify the results obtained by SDS gel electrophoresis in pseudo-native conditions in the presence of a high denaturant concentration, a similar experiment was also carried out for native sfGFP denatured with 6 M GdnHCl. We showed that the presence of the denaturant in the sample does not interfere with the penetration of sfGFP monomers and oligomers into the gel (Supplementary Figure S2A). The SDS gel electrophoresis data for sfGFP fibrils treated with GdnHCl were corroborated by gel filtration experiments. Obtained results indicated the predominant presence in the sample of large protein oligomers with a molecular weight above 300 kDa (Supplementary Figure S2B).

Separation of amyloids’ samples treated with any of the analyzed factor using SDS-PAGE after boiling in 2% SDS for 10 min showed the absence of bands corresponding to the monomeric or oligomeric sfGFP forms on the gel (Supplementary Figure S3). This demonstrates a high resistance to ionic detergents not only of intact fibrils formed from this protein (which is a characteristic feature of amyloids) but also their degradation products.

### 3.4 Analysis of the tertiary structure of aggregates formed during sfGFP amyloid degradation

A unique feature of fibrils formed from sfGFP, which determines their choice as an object of the study, is the presence in their structure of three types of fluorophores: tyrosine residues, a tryptophan residue, and a green chromophore. Three types of internal fluorescent probes make it possible to analyze changes in the structure of amyloids after external influences. It should be noted that the formation of an amyloid fiber from sfGFP is accompanied by a significant reorganization of the secondary and tertiary structures of the native protein.

**FIGURE 2:**
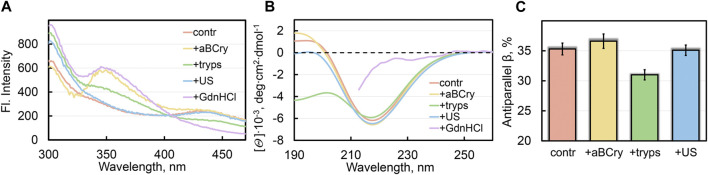
Local and global reorganization of sfGFP amyloid’s structure after their degradation. **(A)** Fluorescence spectra of different fluorophores and **(B)** far-UV CD spectra of sfGFP aggregates. **(C)** Content of β-strands within antiparallel β-sheets in sfGFP aggregates. The colors’ decoding is shown on the panels and corresponds to that on Figures 1F–G.

In particular, among observed changes are the initial melting of three native β-strands, leading to the opening of the protein barrel, the subsequent partial refolding of these strands with the simultaneous unfolding of the central α-helix and the formation from the structural elements disordered at the previous stages of non-native β-strands, which are included in the amyloid fiber (Sulatskaya et al., 2022). These results are in line with data obtained using bioinformatics approaches that define the central α-helix, along with some native β-strands of the protein, as an amyloidogenic segment (Supplementary Table S1). The difference in the structural properties of native sfGFP and its amyloid fiber determines the specific spectral characteristics of their fluorophores. Thus, tyrosine residues (the protein contains 5 Tyr) in sfGFP amyloids are not quenched, in contrast to the native protein, and contribute significantly to amyloids’ UV-fluorescence (Figure 2A, red curve, fluorescence spectrum maximum λ_fl_ = 303 nm). Conversely, the fluorescence of the single tryptophan residue Trp57, which together with the chromophore belongs to the central α-helix in the native protein, is almost completely quenched in sfGFP amyloids. The green chromophore of sfGFP has a distorted structure in the amyloid fiber of the protein, which leads to a blue shift in its absorption and fluorescence spectra (λ_abs_ = 315 nm, λ_fl_ = 435 nm).

All the analyzed factors caused a change in the photophysical characteristics of sfGFP amyloids, as evidenced by the fluorescence spectra of the samples (at the excitation wavelength λ_ex_ = 280 nm) (Figure 2A). The most pronounced changes in the spectral properties of sfGFP fibrils were observed in the presence of GdnHCl (Figure 2A, violet curve). In this sample, the contribution of tyrosine residues to the total UV-fluorescence was more pronounced even compared to intact fibrils. There were also detected an increase in the fluorescence intensity of Trp57 with a red shift of its fluorescence spectrum (maximum at a wavelength of 350 nm), and complete disappearance of the chromophore fluorescence characteristic of intact fibrils. Note that the chromophore itself in sfGFP fibrils treated with GdnHCl was not degraded, since the intensity of its absorption band did not change compared to the control sample of intact fibrils. Therefore, the quenching of the chromophore fluorescence in sfGFP fibrils treated with GdnHCl is rather indicative of a change in the structure of its immediate microenvironment. The Trp57 residue in the thus treated sfGFP fibrils also became available to the solvent, as evidenced by the position of its fluorescence spectrum close to the tryptophan fluorescence maximum in water. A decrease in quenching of tyrosine residues in sfGFP fibrils treated with GdnHCl can be explained by a change in the distances and/or the mutual orientation between them and the Trp57. The chromophore in intact sfGFP fibrils can serve as another acceptor of the excitation energy of tyrosine residues, and as well as the Trp57 residue, given the effective overlap of the absorption spectrum the chromophore and the fluorescence spectra of aromatic residues. Thus, the increased intensity of the fluorescence bands of aromatic residues in the spectrum of sfGFP fibrils treated with GdnHCl is apparently associated with the mutual reorientation of all three types of fluorophores. Considering that both the Trp57 residue and the distorted chromophore belong to the region of the sfGFP polypeptide chain, which is part of the β-sheet that forms the core of the intact amyloid fiber, we can interpret these data in favor of the complete degradation of the fibrillar β-sheet structure of sfGFP fibrils by a denaturing agent.

Treatment of sfGFP fibrils with trypsin led to similar but much less pronounced changes in the fluorescence bands of fluorophores (Figure 2A, green curve) compared to those observed in the presence of GdnHCl. The results obtained confirm the TEM data on the trypsin-induced fragmentation of sfGFP fibrils and partial degradation of the fragmented fiber structure.

sfGFP fibrils exposed to heat shock protein aBCry exhibited the fluorescence bands of the chromophore and tyrosine residues practically unchanged in intensity, while the fluorescence band of the tryptophan residue increased in intensity and red-shifted (maximum at a wavelength of 350 nm) (Figure 2A, yellow curve). These data testify for the local and specific targeting of aBCry to a strictly defined area of the amyloid fiber containing the tryptophan residue, making it completely solvent-exposed. These data are consistent with TEM data showing predominant fluffing of sfGFP fibrils treated with aBCry with the preservation of their core ordering.

Sonication of sfGFP amyloids elicited rather moderate changes in their spectral characteristics compared to the control sample: the intensity and position of the fluorescence spectrum of the tryptophan residue did not change, and the chromophore fluorescence intensity decreased slightly, while a noticeable increase in the fluorescence intensity of tyrosine residues was observed (Figure 2A, blue curve). Preservation of the characteristics of tryptophan residue and chromophore in sfGFP amyloids upon sonication, which are inherent in intact fibrils, indicates maintaining the structure of the fiber core. Together with the TEM data on declustering and fragmentation of sfGFP fibrils treated with ultrasound, we can assume a slight “loosening” of the structure of sfGFP amyloid fragments (possibly at their ends) and the associated change in the orientation of tyrosine residues.

In general, the obtained results indicate that the analyzed external factors affect not only the fibrous morphology and size of amyloids formed from sfGFP, but also their tertiary structure. Again, the observed changes differed depending on the factor applied.

### 3.5 Analysis of the secondary structure of aggregates formed during sfGFP amyloid degradation

The greatest changes in the shape of the circular dichroism (CD) spectrum in the far UV region of sfGFP amyloids occurred after their treatment with GdnHCl and trypsin, in contrast to exposure to ultrasound and aBCry (Figure 2B). The results obtained exclude any pronounced changes in the secondary structure of amyloids after exposure to ultrasound and aBCry. This suggestion was further validated by a quantitative analysis of the recorded CD spectra of the corresponding samples. The amyloids’ sample treated with trypsin had a reduced proportion of β-strands of the antiparallel β-sheet composing the fibril core compared to intact fibrils (Figure 2C). Unfortunately, GdnHCl interferes with collecting the CD spectrum of the sample in the entire wavelength range and prevents its reliable quantitative analysis. Nevertheless, we were able to demonstrate that the CD spectrum of sfGFP amyloids treated with GdnHCl lacked a clearly defined minimum in the region of 220–230 nm (Figure 2B, violet curve), characteristic of proteins enriched in β-sheet structure. This supports our earlier assumption on the complete degradation of the fibrillar core of amyloids by GdnHCl.

Thus, we concluded the different efficiency of the secondary structure loss by sfGFP amyloids under the influence of various external factors. An unexpected result was a similar content of the β-structure forming the amyloid fiber in intact samples and those treated with aBCry and ultrasound, despite the change in the morphology and tertiary structure under these influences.

### 3.6 Analysis of the interaction of a specific ThT fluorescent probe with sfGFP amyloid degradation products

The integrity or, conversely, the destruction of the amyloid fiber induced by external factors was tested by the ability of the degradation products of sfGFP amyloids to bind the specific fluorescent probe thioflavin T (ThT). According to modern concepts, the dye molecule, as well as its derivatives and analogs, are embedded in the grooves formed by the side chains of β-sheet amino acid residues along the axis of the fibril fiber perpendicular to the β-sheets (Krebs et al., 2005; Sulatskaya et al., 2019). This binding model allows the use of ThT for the analysis of the integrity of the amyloid fiber.

**FIGURE 3:**
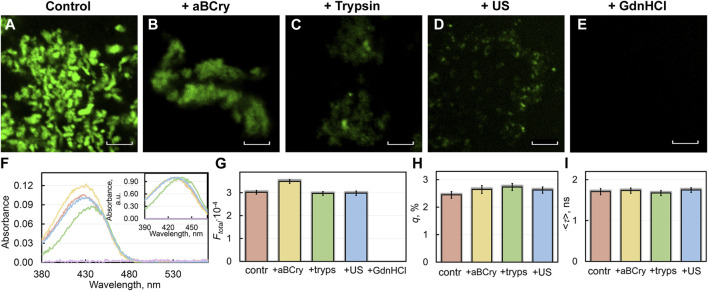
Interaction of sfGFP amyloids before and after degradation with amyloid-specific probe ThT. **(B)** Confocal microscopy of the sfGFP aggregates stained with ThT **(A)** before and **(B–E)** after their treatment by **(B)** aBCry, **(C)** trypsin, **(D)** ultrasound, and **(E)** GdnHCl. The scale bars are equal to 5 μm. **(F)** Absorption spectra, **(G)** integral fluorescence intensity (Ftotal), **(H)** fluorescence quantum yield (q), and **(I)** fluorescence lifetime (<τ>) of ThT bound to sfGFP aggregates. The (Inset) of Panel **(F)** show ThT absorption spectra normalized to unity at the maximum. The colors’ decoding is shown on the panels and corresponds to that on Figures 1F–G.

Confocal microscopy revealed sfGFP aggregates stained with ThT in all studied samples (Figures 3A–D), except for those treated with GdnHCl (Figure 3E). To study the interaction of sfGFP amyloids with the ThT fluorescent probe as well as to evaluate the photophysical characteristics of the fibril-bound dye, we used a specially developed approach (Sulatskaya et al., 2012) based on the spectroscopic study of samples prepared by the equilibrium microdialysis method. Two solutions can be prepared using this approach: a sample solution containing protein aggregates, free and fibril-bound ThT, and a reference solution containing only free ThT in the same solvent and at the same concentration as in the sample solution. This ensures a correct measurement of the absorption spectra of dye molecules associated with amyloids.

A sample of sfGFP fibrils exposed to GdnHCl had near-zero values of absorption (Figure 3F) and total fluorescence intensity (Figure 3G) of fibril-bound ThT. This, in conjunction with confocal microscopy data is coherent with the assumption of the complete degradation of the sfGFP amyloid fiber in the presence of the denaturing agent.

Trypsin treatment of sfGFP fibrils reduced the absorbance of fibril-bound ThT compared to intact amyloids. Close examination of the absorption spectrum of fibril-bound ThT in this case revealed a dip in its left part, and not in the right one. Our previous findings support the existence of two types (modes) of ThT binding to fibrils, with the dye acquiring the absorption spectra different in the positions of the maximum (Kuznetsova et al., 2012). We suspect that a selective reduction in the number of binding sites for one of these modes at the degradation of trypsin-treated amyloids takes place. It has previously been hypothesized that the binding mode of the dye with a lower fluorescence quantum yield (close to that of free ThT in some cases) is associated with its incorporation into the grooves formed by amino acid side chains of β-strands along the long axis of the fibril fiber perpendicular to β-sheets (Krebs et al., 2005). The second mode of the dye binding with a higher fluorescence quantum yield (several orders of magnitude higher than the fluorescence quantum yield of free ThT) is probably attributed to the localization of the dye molecules in the areas of fibrils’ clustering (Sulatskaya et al., 2018). Additionally, the correlation between the position of the dye absorption spectrum and the polarity of its environment has been shown earlier. In particular, the blue-shifted absorption spectrum of free ThT in a highly polar aqueous solution is caused by the strong dipole-dipole interaction of dye and solvent molecules. The shielding the dye from the solvent at binding to amyloid fibers results in a red shift of its absorption spectrum (Sulatskaya et al., 2017a). It can be assumed that the dye incorporated into fibrillar clusters has an even more hydrophobic environment and, accordingly, a more red-shifted absorption spectrum.

Despite the markedly reduced short-wavelength part of the absorption spectrum of ThT bound to trypsin-treated sfGFP amyloids (Figure 3F), the total fluorescence intensity of fibril-bound ThT in the sample remained virtually unchanged (Figure 3G). Taken together, these the data indicate the destruction of only individual amyloid fibers (characterized by a lower fluorescence quantum yield and a blue-shifted absorption spectrum of the bound ThT) under the influence of the above factor, but not amyloid clusters (characterized by a higher fluorescence quantum yield and a red-shifted absorption spectrum of the bound dye). These data are in a good agreement with the results obtained using TEM (Figures 1A–E).

Interestingly, the absorbance and total fluorescence intensity of fibril-bound ThT did not decrease but even increased in the sfGFP amyloids’ sample after exposure to aBCry (Figures 3F,G). In this case, the shape of the absorption spectrum, as well as the lifetime and quantum yield of the bound dye remained almost unchanged (Figures 3H,I). In this regard, it can be assumed that the “fluffing” of sfGFP regions outside the fibrillar core increased the accessibility of both types of dye binding sites and, in turn, led to an increase in the number of bound dye molecules.

All spectral characteristics of the fibril-bound ThT measured for the sfGFP amyloids’ sample before and after sonication, including absorbance, total fluorescence intensity, fluorescence quantum yield, and fluorescence lifetime, as well as the shape and position of the absorption spectrum, were almost the same (Figures 3F–I). These data argue for the identity of amyloid fibrils in the treated and control samples. These results are in good agreement with the TEM data on the invariance of morphology, as well as with a slight change in the intrinsic spectral characteristics and secondary structure of amyloid fibers after such exposure.

### 3.7 Cytotoxicity of aggregates formed during degradation of SfGFP amyloids by external factors

**FIGURE 4:**
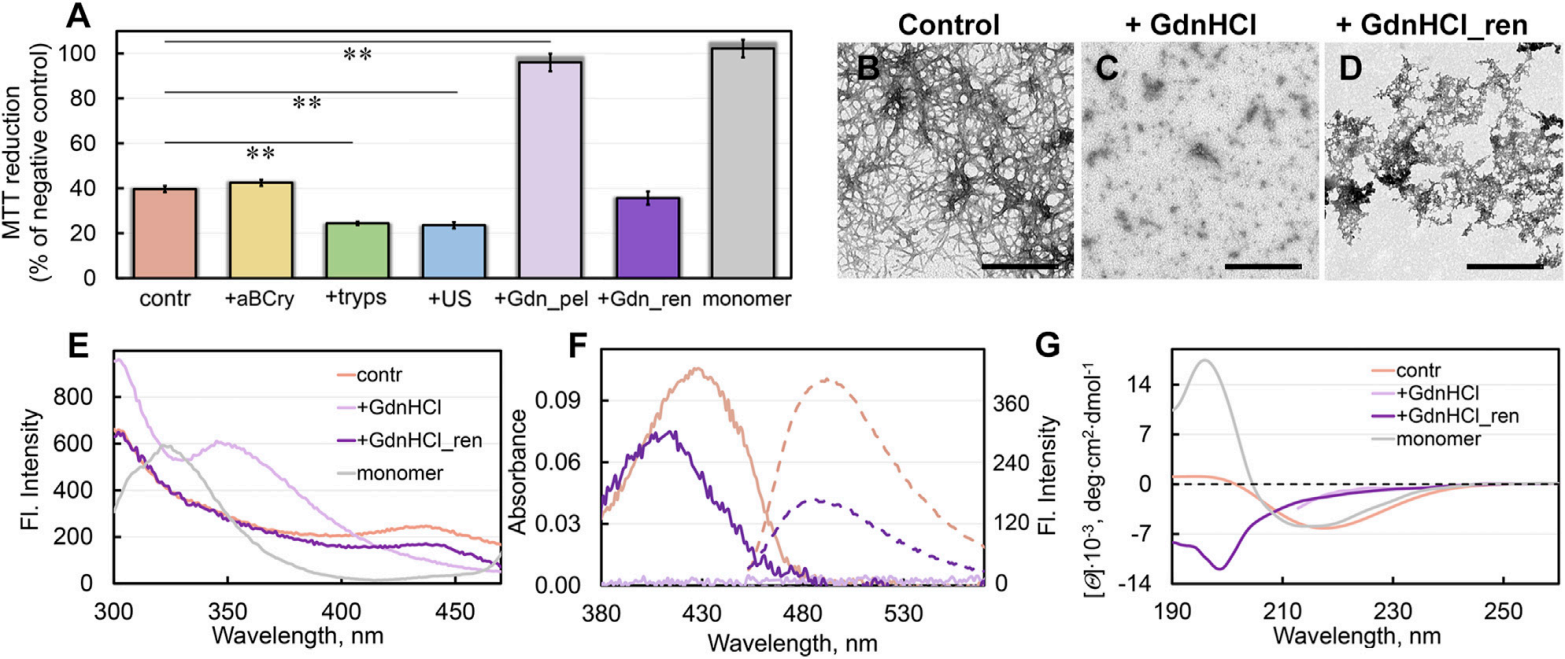
Cytotoxicity of sfGFP fibrils before and after degradation and their repolymerization after GdnHCl exposure. **(A)** Inhibition effect of sfGFP amyloids at concentration 0.1 μM on HeLa cells determined by MTT assay. Cells were exposed to aggregates, intact (contr) and after their treatment by aBCry, trypsin (tryps), and ultrasound (US). Data are also presented for the cells exposed to aggregates from pellet after centrifugation of sfGFP amyloids treated by GdnHCl (Gdn_pel) and to reassembled aggregates after denaturant removal from the sample (Gdn_ren). Cell viability after exposure to a monomeric sfGFP (monomer) is shown as a control. Data are expressed as mean ± SEM based on triplicate samples. **p < 0.05 (n = 5, ANOVA, Tukey’s *post hoc* test). **(B–D)** The electron micrographs of sfGFP aggregates **(B)** intact, **(C)** after their treatment by GdnHCl, and **(D)** after denaturant removal from the sample. Scale bars are equal to 500 nm. **(E)** Fluorescence spectra of different fluorophores and **(F)** far-UV CD spectra of sfGFP aggregates before (contr) and after GdnHCl exposure, after denaturant removal from the sample (GdnHCl_ren) and native monomeric sfGFP (monomer). **(G)** Absorption (solid lines) and fluorescence (dotted lines) spectra of ThT bound to sfGFP aggregates before and after GdnHCl exposure and after denaturant removal from the sample. The colors’ decoding is shown on panels **(E)** and **(G)**.

We analyzed the impact of the observed changes in the morphology and structure of sfGFP amyloids after exposure to various factors on fibrils cytotoxicity (Figure 4A). We estimated the viability of HeLa cells cultured in the presence of various fibril degradation products during the day. Given the fact that protein aggregates after exposure to external factors to a large extent retained features typical of amyloid, it could be expected that they also retained high cytotoxicity. The results obtained are in good agreement with this assumption. Fibrils “fluffed up” by aBCry, as well as predominantly fragmented with trypsin or ultrasound, were not less cytotoxic than intact amyloids. Moreover, we surprisingly found an almost 2-fold higher cytotoxicity of fibrils treated with trypsin and ultrasound (at an amyloid concentration of about 0.5 μg/mL) compared to the control sample. As reported in literature (Mannini et al., 2014; Limbocker et al., 2023), there is a correlation between the aggregate cellular toxicity on one hand and their size and surface hydrophobicity on the other. It can be noted that the size of the protein species in the samples after exposure to trypsin or ultrasound, which had a greater toxicity to cells, was indeed smaller according to the TEM data and the values of RLS and turbidity. We also characterized the solvent-exposed hydrophobic regions by 1-anilino-8-naphthalene sulfonate (ANS) dye fluorescence measurements (Mannini et al., 2014). This hydrophobic probe has low fluorescence quantum yield in polar environments, such as aqueous solutions, but its fluorescence is dramatically increased in nonpolar environments (see, e.g., (Mendoza et al., 1991; Semisotnov et al., 1991; Gasymov and Glasgow, 2007; Walchli et al., 2020)). Thereby, an increase in fluorescence intensity of ANS is generally assumed to reflect its binding to hydrophobic regions in proteins. Data on the dye fluorescence intensity in untreated and treated with various factors suspensions of amyloids (Supplementary Figure S4, samples for analysis were prepared by equilibrium microdialysis) fit into the previously proposed correlation between the cytotoxicity of protein aggregates and the hydrophobicity of their surface.

Unfortunately, we were unable to assess the cytotoxicity of the sample treated with GdnHCl, because of the extreme toxicity to cells of the denaturing agent itself. However, we determined the cytotoxicity of the denaturant-free pellet after centrifugation (18,000 rpm, 2 h) of a sample treated with GdnHCl (Figure 4A). Thus, obtained sample practically did not reduce cell viability. Based on our experimental findings we can propose that the treatment of sfGFP amyloids with GdnHCl induce ether 1) the transformation of large toxic intact amyloids into large non-toxic protein aggregates, or 2) the decomposing of large amyloid clots into smaller aggregates or individual fibers that cannot be precipitated by centrifugation. To find out which of the hypotheses is correct, we analyzed the absorption of the pellet collected by centrifugation. The protein absorption spectrum was barely detectable in the pellet, indicating the absence of large protein aggregates in the GdnHCl-treated amyloids’ sample. These data are in good agreement with second hypothesis postulating the GdnHCl-induced degradation of the large toxic amyloid clots present in the intact sample.

### 3.8 Repolymerization of sfGFP amyloids degraded by GdnHCl

We also traced the structural transformations of sfGFP amyloid degradation products after the removal of GdnHCl from the sample by dialysis. The sfGFP amyloid sample pre-treated with GdnHCl was incubated after removal of the denaturant under conditions required for spontaneous fibrillogenesis of the native protein. Note that the sample did not acquire a specific green color after 3 weeks of incubation. This points at the inability of the polypeptide chain to refold into a native β-barrel with the green chromophore inside under these conditions.

The sample of sfGFP amyloid degradation products upon incubation after removal of GdnHCl became distinctly opalescent. This implies an increase in the linear dimensions of aggregates in the sample. The TEM data corroborated this assumption by showing the formation of large aggregates without a pronounced fibrous structure in the sample (Figures 4B–D). Spectral analysis of these aggregates showed the recovery of the chromophore fluorescence at 435 nm and tyrosine fluorescence lost at disassembly of sfGFP amyloids by GdnHCl up to 70% and 95% of the level for intact amyloid fibrils of the protein, respectively (Figure 4E).

Additionally, removal of GdnHCl from the sample of degraded sfGFP amyloids restored the absorbance and the total fluorescence intensity of bound to them ThT up to 70% and 40% of the level for intact fibrils, respectively (Figure 4F). At the same time, the absorption spectrum of the dye bound to newly-formed aggregates of sfGFP differed markedly the shape and position (maximum at about 420 nm) from the spectrum of the ThT embedded in fibrils (maximum at about 430–450 nm). We have observed previously a slightly red-shifted absorption spectrum of the dye (with a maximum at about 420–425 nm) relative to the absorption spectrum of the free dye in a dilute aqueous solution (with a maximum at about 412 nm) in the case of its binding to the hydrolytic enzyme acetylcholinesterase, as well as in solvents with high viscosity or low solvent polarity. Thus, observed in our case photo-physical properties of ThT are not compatible with its incorporation into amyloid fibrils, but are likely explained by less specific interactions.

To assess the secondary structure of the reassembled fibrils, we recorded their far-UV CD spectrum. This spectrum has nothing in common with either the spectra of intact fibrils or the spectra of a monomeric native protein (Figure 4G). A quantitative analysis showed that the newly-formed sfGFP aggregates had a relatively high content of the antiparallel β-sheet structure (more than 30%) (Supplementary Figure S5). In this regard, we can relate the binding of the dye to these reassembled aggregates to the partial formation β-sheet core in them. At the same time, the reassembled sfGFP aggregates lacked the structural elements characteristic of mature fibrils from this protein: α-helices and β-strands arranged in a parallel β-sheets. Instead, they contained an increased content of disordered elements and β-turns compared to mature amyloids. Apparently, the aggregates reassembled after sfGFP amyloids degradation contained no the secondary structure characteristic of intact amyloids, both in the amyloid fiber itself and outside the fibrillar core.

The question of the cytotoxicity of the newly formed aggregates turned out to be especially intriguing. It turned out that these aggregates not only have similar physicochemical properties with amyloids, but also have an equally high cytotoxicity (Figure 4A). Based on all our data, the aggregates, formed after the preliminary degradation of sfGFP fibrils with GdnHCl and subsequent removal of the denaturant from the sample, can be regarded as amyloid-like ones.

### 3.9 Degradation of pathological amyloids formed from Aβ-peptide

The universality of the revealed influence of the analyzed external factors on the morphology and cytotoxicity of amyloids was tested on the example of pathological amyloids formed from the Aβ-peptide with a length of 42 amino acids (Aβ42). Accumulation of Aβ42 fibrils as amyloid plaques accompanies Alzheimer’s disease.

**FIGURE 5:**
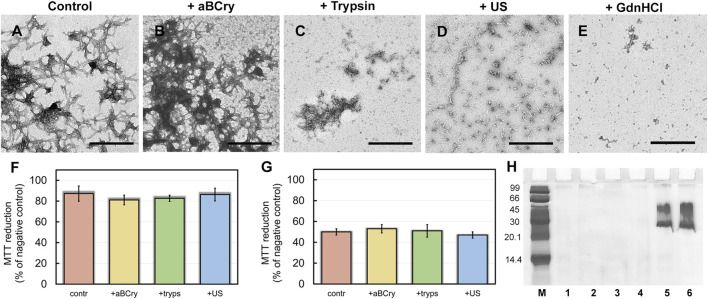
Aβ42 amyloid fibrils degradation. The electron micrographs of Aβ42 aggregates **(A)** before and **(B–E)** after their treatment by **(B)** aBCry, **(C)** trypsin, **(D)** ultrasound, and **(E)** GdnHCl. The scale bars are equal to 500 nm. **(F,G)** Inhibition effect of Aβ42 amyloids on HeLa cells determined by MTT assay. Cells were exposed to aggregates, intact (contr) and after their treatment by aBCry, trypsin (tryps), and ultrasound (US) at concentrations **(F)** 0.015 and **(G)** 0.075 μM. Data are expressed as mean ± SEM based on triplicate samples. **(H)** Native PAGE (17% gel) of Aβ42 aggregates before (lane 1) and after their treatment by trypsin (lane 2), GdnHCl (lane 3), ultrasound (lane 4), and aBCry (lane 5). (lane 6) corresponds to aBCry alone. **(M)** corresponds to marker proteins.

The degradation of Aβ42 amyloids followed remarkably similar mechanisms to that of sfGFP amyloids when treated with the same factors. Short fibers of Aβ42 present in the control sample, prone to clustering (Figure 5A), underwent: 1) partial “fluffing” and “disordering” practically without size reduction in the presence of aBCry (Figure 5B), 2) preferential degradation, with highly clustered areas unaffected, in the presence of trypsin (Figure 5C), 3) declustering and fragmentation by sonication (Figure 5D), and 4) degradation to small amorphous aggregates by GdnHCl (Figure 5E). As with sfGFP amyloids, none of the interventions could suppress the cytotoxicity of Aβ42 amyloids (Figures 5F,G). This result is probably due to the fact that for all factors we observed only a change in the morphology and sizes of aggregates, and not thecomplete destruction of amyloids into monomeric subunits (Figure 5H) necessary for reducing cytotoxicity. Given that a decrease in the size and an increase in the hydrophobicity of amyloid aggregates surface leads to an increase in their cytotoxicity (Mannini et al., 2014; Limbocker et al., 2023), incomplete degradation, including accompanied by fragmentation of amyloids, will lead either to 1) the preservation of cytotoxicity (if the fragments do not critically change their size and hydrophobicity) or 2) to its increase (if the change in these parameters is significant). The choice of the implemented scenario depends on the specific factor and properties of amyloid fibrils (amyloid proteins).

## 4 Discussion

In the present study, we analyze the resistance of model amyloids formed from sfGFP and pathological amyloids formed from Aβ42 to a wide range of external factors. It should be remembered that protein aggregates not only from different proteins, but also from the same protein, formed under different conditions, can have very different morphology and properties (Sulatskaya et al., 2017b; Stepanenko et al., 2021b; Limbocker et al., 2023). Nevertheless, two randomly selected amyloids with different sizes, structures, and properties exhibited similar mechanisms of degradation under the influence of various external factors. We have shown that all the factors applied are capable of remodeling amyloids via different mechanisms. In particular, the following schemes of amyloid degradation have been demonstrated: local “fluffing” of the structure of amyloids without disturbing of their fibrillar core and reducing the size (aBCry); declustering of amyloid clots and fragmentation of fibrils without changing their secondary structure (ultrasound); fibril fiber fragmentation and partial “fluffing” of the fragmented pieces without declustering of amyloid clots (trypsin); disintegration of amyloid clots and complete degradation of the fibrillar β-sheet core with the formation of high molecular weight oligomers (GdnHCl). The described here mechanisms of sfGFP amyloids degradation by external factors are consistent with the earlier literature data for fibrils formed from other amyloidogenic proteins and peptides (including those involved in the pathogenesis of various amyloidoses) (Narimoto et al., 2004; Okumura and Itoh, 2014; Poepsel et al., 2015; Stepanenko et al., 2020; Sulatsky et al., 2020; Stepanenko et al., 2021a). This argues for the universality of the observed effects. The use of sfGFP fibrils as an object of study enabled to detect local and global changes in the packing of amino acid residues in an amyloid fiber under the influence of external factors. The extreme sensitivity of the spectral characteristics of intrinsic sfGFP fluorophores to changes in the morphology of fibrils formed from this protein justifies the applicability of sfGFP amyloids as a model test object.

**FIGURE 6:**
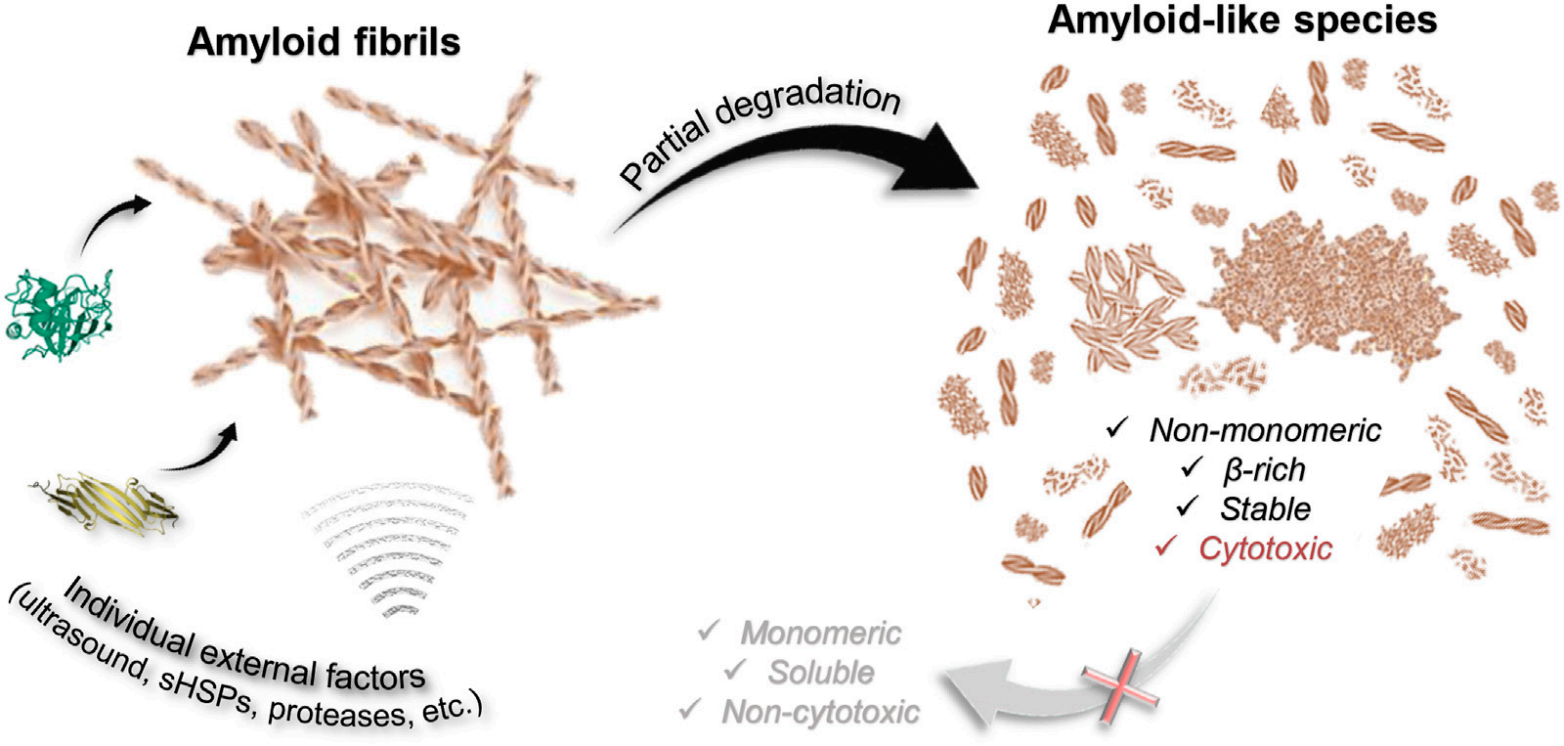
Scheme illustrating that degradation of amyloids induced by some external factors of various nature (including chemical and mechanical stresses) is partial. None of the considered factors (aBCry, trypsin, ultrasound, SDS, boiling, boiling in the presence of SDS) ensures complete depolymerization of amyloids to monomeric subunits. At the same time, almost all the polymorphic structures obtained as a result of degradation retain the properties inherent in amyloids: high stability, high content of β-strands, specific binding to ThT, and most importantly, identical to intact amyloids or even higher toxicity to cells.

The first important finding of the current study is that most factors are unlikely to destroy a highly stable amyloid fiber completely (Figure 6). Of the factors analyzed by us, only GdnHCl had relatively strong effect, however, insufficient for the depolymerization of amyloids into individual monomers, oligomers, and aggregates smaller than 200 kDa. At the same time, we noted the dependence of the efficiency of fibril degradation using other factors (most clearly demonstrated by trypsin) on the degree of fiber clustering: amyloids, which were large fibrillar clots, were difficult to destroy. This can be attributed to the inaccessibility of binding and action sites of external factors, as well as the presence of additional intermolecular bonds that need to be broken.

A wide range of species described and analyzed in current work is potentially identical to those structures accumulating during the degradation of toxic amyloids in the body. Almost all the polymorphic structures formed as a result of amyloid degradation have been shown to retain some of their inherent properties (Figure 6). In addition to the aforementioned preserved β-sheet structure of the fibrillar core of most of the products of amyloid degradation, they were also highly resistant to ionic detergents and boiling. At the same time, as discussed above, even GdnHCl the most effective of the analyzed factors fails to destroy fibrils to monomers or even small oligomers that could be easily eliminated from the body by cell defense systems.

Importantly, we have also found that the cytotoxicity of fragmented and/or decompacted fibrils may not only not decrease relative to intact amyloids (in the case of exposure to aBCry), but also significantly increase (in the case of exposure to trypsin and ultrasound on sfGFP amyloids) (Figure 6). These results are in good agreement with the literature data (Xue et al., 2009; Bystrenova et al., 2019; Sulatsky et al., 2020; Stepanenko et al., 2021a). In particular, there are ideas about damage to the cell membrane by fragmented fibril and the resulting increase in the cytotoxic effect of amyloids. In addition, decompacted fibrils may have an increased affinity for cell membranes, resulting in their excessive stabilization or destabilization (Cheng et al., 2013). Thus, it turned out that none of analyzed external factors, which do not actually affect cell viability, can reduce the cytotoxicity of amyloids, despite their degradation (Figure 6). At the same time, it was shown that even if a decrease in cytotoxicity under the influence of some factor is possible, this effect would come to naught as a result of the repolymerization of aggregates after the removal of the influence (exemplified by GdnHCl).

Another important finding of the work is that even the process of rather efficient degradation of fibrils after elimination of the degrading influence can be reversed into reaggregation of amyloidogenic proteins (exemplified by GdnHCl). This finding is in good agreement with the previous data (Narimoto et al., 2004; Sulatsky et al., 2020). However, the fundamental novelty of current result is underscored by the discovery of altered structure of reassembled sfGFP amyloid-like aggregates compared to intact amyloids. Indeed, the newly formed sfGFP aggregates lacked a fibrous morphology of amyloids. This contrasts to some extent with the results of our previous studies on the complete restoration of the structure and properties of fibrils for amyloids formed from other amyloidogenic proteins after elimination of the degrading influence (Sulatsky et al., 2020). It may underline the fundamental importance for assembly and reassembly of *bona fide* amyloids from β-barrel proteins, including sfGFP, of their original native structure, which, as shown here, is not restored under conditions of fibrillogenesis after amyloid treatment with GdnHCl. This assumption is supported by our early results considering a highly structured intermediate state of sfGFP with a largely preserved structure of the native β-barrel as a driver for its aggregation along the pathway of amyloid fibrils formation (Sulatskaya et al., 2022). Moreover, the realization of alternative pathways of sfGFP aggregation with the formation of, for example, amorphous aggregates (Stepanenko et al., 2021b) is obviously associated with the formation of various intermediate states during protein denaturation. Collectively, our current and previous results (Stepanenko et al., 2021b) reveal a relationship between the structure of formed protein aggregates (both amyloid fibrils and amyloid-like or amorphous aggregates) and the structure of the intermediate state.

Our results are also an important step towards solving the urgent problem of drug treatment of amyloidosis. We show that therapeutic agents aimed at degradation of amyloid plaques should be used with extreme caution. According to the results of the work and literature data (Emeson et al., 1966; Soderberg et al., 2005; Chander et al., 2006; Shin et al., 2008; Giorgetti et al., 2011; Azevedo et al., 2012; Gao et al., 2015; Poepsel et al., 2015; Yagi et al., 2015; Sulatsky et al., 2020; Stepanenko et al., 2021a), most potential drugs aimed at removing amyloid from the body can only destroy individual amyloid fibers, and not dense amyloid plaques. At the same time, unpredictable consequences of incomplete degradation of fibrils can aggravate the patient’s condition due to amplification of fibrillogenesis, the rapid spread of amyloid seeds between cells as well as increased cytotoxicity of the products of amyloid disassembly (Xue et al., 2008; Xue et al., 2009; Xue et al., 2010; Mannini et al., 2014). Similar results, indicating a possible rise in the amyloids’ cytotoxicity during and after their degradation under the influence of external factors were obtained by other authors (Giorgetti et al., 2011; Yagi et al., 2015). Thus, specific attention in the search for potential therapeutic agents should be focused on the study of the rate of degradation of amyloids and the cytotoxicity of intermediate species accumulated in this process.

## 5 Conclusion

The results of a comprehensive analysis of the degradation of cytotoxic amyloids induced by chemical and mechanical factors show that the mechanism of fibril degradation depends on the applied external factor rather than the type of amyloids. Individual external factors often provide only partial degradation of amyloids without leading to the formation of monomeric soluble subunits. Regardless of the method of fibril degradation, the resulting fragments retain some amyloid’s properties, may have even higher cytotoxicity than mature fibrils, and, according to the literature, serve as a seed for the formation of new amyloid fibrils (Xue et al., 2008; Xue et al., 2009; Xue et al., 2010). The results of our work indicate that the degradation of amyloid fibrils *in vivo* should be treated with caution since such an approach can lead not to recovery, but to aggravation of the disease.

Our results suggest that a truly effective and safe treatment strategy for amyloidosis should include multiple active agents with different mechanisms of action. Among them are the agents that consistently lead to: 1) declustering of amyloid plaques into individual fibers, 2) rapid depolymerization of fibrils from the ends without their fragmentation, 3) denaturation of monomeric proteins cleaved from the fibrillar core, 4) degradation of denatured proteins and their elimination from the body. The last step is particularly crucial, given that the development of a drug providing quick and effective fibril degradation is insufficient. Aggregates of partially unfolded proteins that remain unexcreted in the body will be prone to rapid repolymerization into amyloids with identical or altered structure and properties immediately after a decrease in the concentration of the therapeutic agent in the body (as in the case of GdnHCl). Last but not least, our results highlight the applicability of amyloids of the fluorescent protein sfGFP as a suitable model object for the search, development, and testing of drugs, since their internal fluorophores serve as sensors of the transformations of the amyloid-forming protein during fibrils’ degradation.

## Data Availability

The original contributions presented in the study are included in the article/Supplementary Material, further inquiries can be directed to the corresponding author.
