# Supplementary material for: Amyloid fibrils degradation: the pathway to recovery or aggravation of the disease?
Source: Front Mol Biosci. 2023 Jun 12;10:1208059. doi: 10.3389/fmolb.2023.1208059 (PMC10291066; doi:10.3389/fmolb.2023.1208059)
Supplement: Supplementary file 1 [file Image5.pdf]

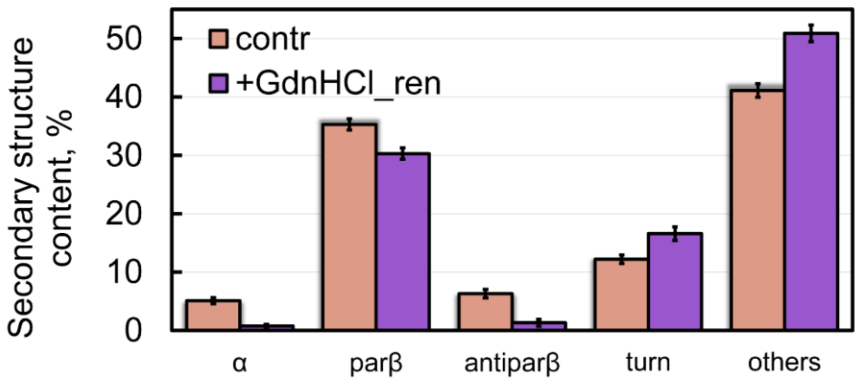

Supplementary Figure 5. Deconvolution of the CD spectra of sfGFP aggregates using the BeStSel method (1). Data are presented for intact sfGFP amyloids (contr) and sfGFP aggregares formed after treatment of amyloids with GdnHCl and removing denaturant from the sample (GdnHCl_ren). Here we represent the change in the content of α-helices (α), β-strands within parallel (parβ) and antiparallel (antiparβ) β-sheet, β-turns (turn), and other structures (includes 3-10-helices, bends, and unordered structure).
