# Supplementary material for: Amyloid fibrils degradation: the pathway to recovery or aggravation of the disease?
Source: Front Mol Biosci. 2023 Jun 12;10:1208059. doi: 10.3389/fmolb.2023.1208059 (PMC10291066; doi:10.3389/fmolb.2023.1208059)
Supplement: Supplementary file 2 [file Table1.pdf]

Supplementary Table 1. The amyloidogenic segments[1] of sfGFP predicted by on-line methods.

amino acid sequence and structural elements[2]		AGGRESCAN (2)	PASTA 2.0 (3)	WALTZ (4)	Tango (5)
	M				
	S				
	K				
	G				
	E				
α	E				
	L		L		
	F	F	F		
	T	T	T		
	G	G	G		
	V	V	V		
	V	V	V		
	P		P		
	I	I	I		
	L	L	L		
β1	V	V	V		
	E	E			
	L	L			
	D				
	G				
	D				
	V				
	N				
	G				
	H				
	K				
	F				
	S				
	V				
β2	R				
	G				
	E				
	G				
	E				
	G				
	D				
	A				
α	T				
	N				
	G				
	K				
	L	L			
	T	T			
	L	L			
β3	K	K			
	F	F			
	I	I			
	C	C			
	T	T			
	T	T			
	G	G			
	K	K			
	L	L			

amino acid sequence and structural elements[2]		AGGRESCAN (2)	PASTA 2.0 (3)	WALTZ (4)	Tango (5)
	P				
	V				
	P				
	W				
	P				
	T	T	T		T
	L	L	L		L
	V	V	V		V
	T	T	T		T
	T	T	T		T
	L	L	L		L
	**CRO**	X	X		X
	V	V	V		V
	Q	Q	Q		Q
	C	C	C		C
central α	F	F	F		F
	S	S	S		
	R		R		
	Y		Y		
	P				
	D				
	H				
	M				
	K				
	R				
	H				
	D				
	F				
	F				
	K				
	S				
	A				
	M				
	P				
	E				
	G				
	Y				
	V				
β4	Q				
	E				
	R				
	T				
	I				
	S				
	F				
	K				
	D				
	D				
	G				
	T				
	Y				
β5	K				
	T				
	R				
	A				
	E				

amino acid sequence and structural elements[2]		AGGRESCAN (2)	PASTA 2.0 (3)	WALTZ (4)	Tango (5)
	V				
	K				
	F				
	E				
	G				
	D				
	T				
	L				
	V				
β6	N				
	R				
	I				
	E				
	L				
	K				
	G				
	I				
	D				
	F				
	K				
	E				
	D				
	G				
	N				
	I				
	L				
	G				
	H				
	K				
	L			L	
	E			E	
	Y		Y	Y	
	N		N	N	
	F		F	F	
	N		N	N	
	S	S	S		
	H	H	H	H	
β7	N	N	N	N	
	V	V	V	V	V
	Y	Y	Y	Y	Y
	I		I	I	I
	T		T	T	T
	A			A	A
α	D				
	K				
	Q				
	K				
	N				
	G				
	I				
β8	K				
	A				
	N				
	F				
	K				
	I				

amino acid sequence and structural elements[2]		AGGRESCAN (2)	PASTA 2.0 (3)	WALTZ (4)	Tango (5)
	R				
	H				
	N				
	V				
	E				
	D				
	G				
	S				
	V				
	Q				
	L				
β9	A				
	D				
	H				
	Y				
	Q				
	Q				
	N				
	T				
	P				
	I				
	G				
	D				
	G				
	P				
	V				
	L				
	L				
	P				
	D				
	N				
	H				
	Y				
β10	L				
	S				
	T				
	Q				
	S				
	V				
	L				
	S				
	K				
	D				
	P				
	N				
	E				
	K				
	R				
	D				
	H				
β11	M				
	V	V		V	
	L	L		L	
	L	L		L	
	E	E		E	
	F	F		F	F

amino acid sequence and structural elements[2]	AGGRESCAN (2)	PASTA 2.0 (3)	WALTZ (4)	Tango (5)
V	V		V	V
T	T		T	T
A	A		A	A
A	A			A
G	G			G
I	I			I
T				
H				
G				
M				
D				
E				
L				
Y				
K				
G				
S				

[1] Segments of the protein sequence that predicted to be amyloidogenic are indicated by gray boxes.
[2] Regions of the protein sequence belonging to α-helixes and β−strands are highlighted in pink and blue, respectively.

1 References

1. A. Micsonai, F. Wien, E. Bulyaki, J. Kun, E. Moussong, Y. H. Lee, Y. Goto, M. Refregiers and J. Kardos: BeStSel: a web server for accurate protein secondary structure prediction and fold recognition from the circular dichroism spectra. *Nucleic Acids Res*, 46(W1), W315-W322 (2018) doi:10.1093/nar/gky497

2. O. Conchillo-Sole, N. S. de Groot, F. X. Aviles, J. Vendrell, X. Daura and S. Ventura: AGGRESCAN: a server for the prediction and evaluation of "hot spots" of aggregation in polypeptides. *BMC Bioinformatics*, 8, 65 (2007) doi:10.1186/1471-2105-8-65

3. I. Walsh, F. Seno, S. C. Tosatto and A. Trovato: PASTA 2.0: an improved server for protein aggregation prediction. *Nucleic Acids Res*, 42(Web Server issue), W301-7 (2014) doi:10.1093/nar/gku399

4. S. Maurer-Stroh, M. Debulpaep, N. Kuemmerer, M. Lopez de la Paz, I. C. Martins, J. Reumers, K. L. Morris, A. Copland, L. Serpell, L. Serrano, J. W. Schymkowitz and F. Rousseau: Exploring the sequence determinants of amyloid structure using position-specific scoring matrices. *Nat Methods*, 7(3), 237-42 (2010) doi:10.1038/nmeth.1432

5. A. M. Fernandez-Escamilla, F. Rousseau, J. Schymkowitz and L. Serrano: Prediction of sequence-dependent and mutational effects on the aggregation of peptides and proteins. *Nat Biotechnol*, 22(10), 1302-6 (2004) doi:10.1038/nbt1012
