# Supplementary material for: Amyloid fibrils degradation: the pathway to recovery or aggravation of the disease?
Source: Front Mol Biosci. 2023 Jun 12;10:1208059. doi: 10.3389/fmolb.2023.1208059 (PMC10291066; doi:10.3389/fmolb.2023.1208059)
Supplement: Supplementary file 3 [file Image4.pdf]

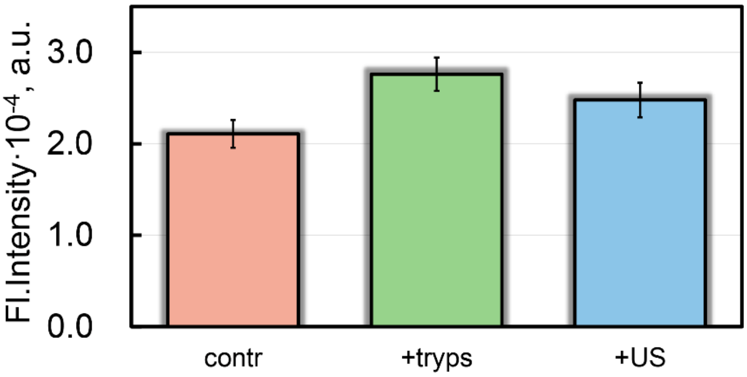


**Supplementary Figure 4.** Analysis of the solvent-exposed hydrophobic regions of sfGFP amyloids by 1-anilino-8-naphthalenesulfonate (ANS) dye fluorescence measurements. Total ANS fluorescence in suspensions of untreated amyloids (contr) and amyloids treated with trypsin (+tryps) and ultrasound (US) are presented. Samples for analysis were prepared by equilibrium microdialysis.
