# Supplementary material for: Amyloid fibrils degradation: the pathway to recovery or aggravation of the disease?
Source: Front Mol Biosci. 2023 Jun 12;10:1208059. doi: 10.3389/fmolb.2023.1208059 (PMC10291066; doi:10.3389/fmolb.2023.1208059)
Supplement: Supplementary file 4 [file Image2.pdf]

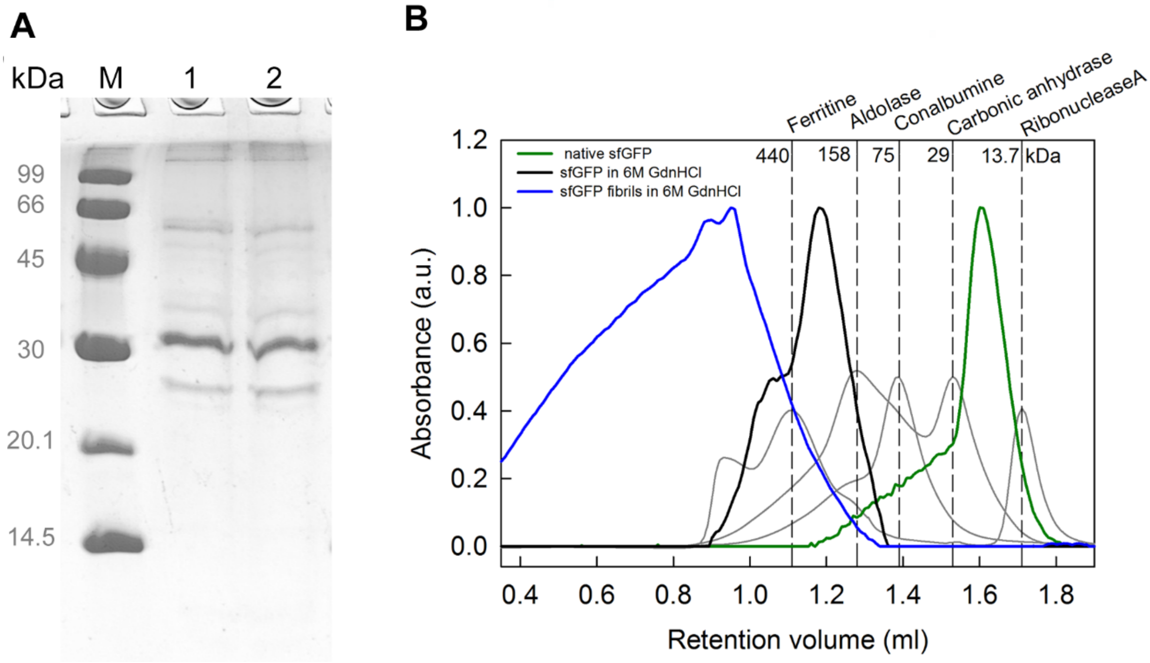

Supplementary Figure 2. Evaluation of the size of the species formed as a result of sfGFP fibrils degradation after guanidine hydrochloride (GdnHCl) exposure. (A) The sfGFP treated with 6 M GdnHCl (lane 1) and additionally boiled (lane 2) were analyzed by sodium dodecyl sulfate (SDS) gel electrophoresis in pseudo-native conditions. Marker proteins (M) of known molecular weight are shown. (**B**) The elution profile of sfGFP in native conditions recorded at absorption wavelength of 280 nm (green line) had a main peak corresponding to the monomeric state of sfGFP. The elution profile of denatured monomeric sfGFP (black line) was shifted toward smaller elution volumes. In addition, this elution profile had a pronounced shoulder preceding the main peak, which probably corresponds to the oligomeric sfGFP forms, which we also observed using SDS gel electrophoresis in pseudo-native conditions. The sample of sfGFP fibrils exposed to GdnHCl (blue line) eluted with a broad split peak with a void volume of the column. This is consistent with the high molecular weight heterogeneous oligomeric form of the protein. The protein molecular weight standards (gray lines) were used to calibrate the column under native conditions.
