# Supplementary material for: Amyloid fibrils degradation: the pathway to recovery or aggravation of the disease?
Source: Front Mol Biosci. 2023 Jun 12;10:1208059. doi: 10.3389/fmolb.2023.1208059 (PMC10291066; doi:10.3389/fmolb.2023.1208059)
Supplement: Supplementary file 5 [file Image3.pdf]

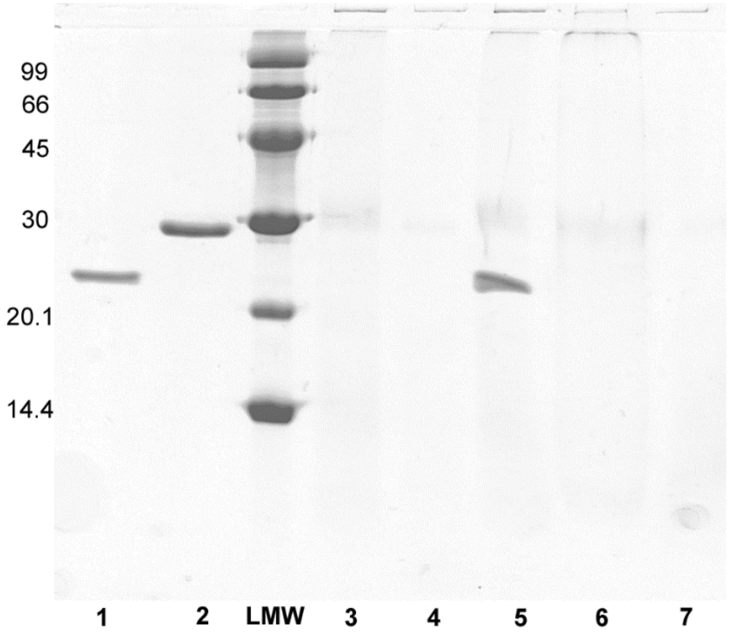

Supplementary Figure 3. SDS-PAGE of aggregates after their boiling in 2 % SDS. On a 17 % gel sfGFP were loaded: (lane 1) aBCry alone; (lane 2) monomeric sfGFP; (LMW) low molecular weight marker proteins; aggregates before (lane 3) and after their treatment with trypsin (lane 4), aBCry (lane 5), GdnHCl (lane 6), and ultrasound (lane 7).
