# Supplementary material for: Amyloid fibrils degradation: the pathway to recovery or aggravation of the disease?
Source: Front Mol Biosci. 2023 Jun 12;10:1208059. doi: 10.3389/fmolb.2023.1208059 (PMC10291066; doi:10.3389/fmolb.2023.1208059)
Supplement: Supplementary file 6 [file Image1.pdf]

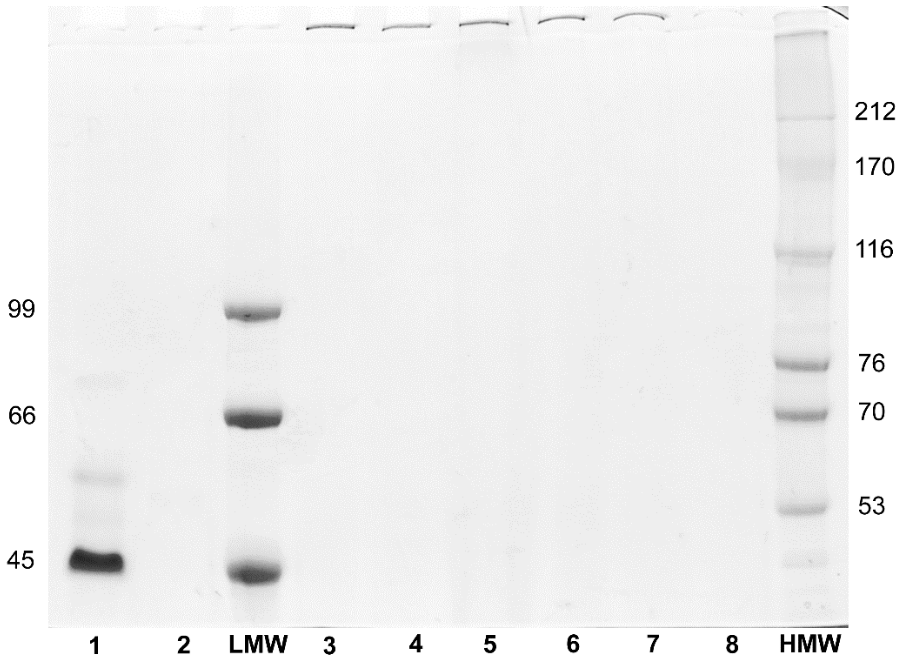

Supplementary Figure 1. Pseudo-native SDS-PAGE. On a 8 % gel were loaded: (lane 1) and (lane 2) monomeric sfGFP before and after boiling, respectively; (LMW) low molecular weight marker proteins; aggregates before (lane 3) and after their treatment with trypsin (lane 4), GdnHCl (lane 5), ultrasound (lane 6), and aBCry (lane 7); (lane 8) aBCry alone; (HMW) high molecular weight marker proteins.
